# Memory in Social Interactions: The Effects of Introspection on Destination Memory in Traumatic Brain Injury

**DOI:** 10.3390/brainsci13091250

**Published:** 2023-08-27

**Authors:** Mohamad El Haj, Ahmed A. Moustafa, Philippe Allain

**Affiliations:** 1 Institut Universitaire de France, F-75005 Paris, France; 2 CHU Nantes, Clinical Gerontology Department, Bd Jacques Monod, F-44093 Nantes, France; 3 LPPL-Laboratoire de Psychologie des Pays de la Loire, Faculté de Psychologie, Université de Nantes, Chemin de la Censive du Tertre, BP 81227, CEDEX 03, F-44312 Nantes, France; 4 School of Psychology, Faculty of Society and Design, Bond University, Gold Coast, QLD 4229, Australia; 5 Department of Human Anatomy and Physiology, The Faculty of Health Sciences, University of Johannesburg, Johannesburg 2006, South Africa; 6 Laboratoire de Psychologie des Pays de la Loire, LPPL EA 4638, SFR Confluences, UNIV Angers, Maison de la recherche Germaine Tillion, 5 bis Boulevard Lavoisier, CEDEX 01, F-49045 Angers, France; 7 Département de Neurologie, Centre Hospitalier Universitaire d’Angers, F-49000 Angers, France

**Keywords:** destination memory, introspection, social cognition, traumatic brain injury (TBI)

## Abstract

Destination memory, which is the ability to remember to whom one has sent information, is intimately associated with social cognition. We assessed whether processing attributes of destinations would improve destination memory in patients with traumatic brain injury (TBI). In this cross-sectional study, we tested the destination memory of 24 patients with TBI and 25 control participants in two conditions. On the first one (control condition), we invited participants to tell proverbs to celebrities’ faces in order to decide, on a subsequent recognition test, whether they previously told that proverb to that celebrity or not. On the second condition (experimental introspection condition), the same procedures were repeated. However, after telling the proverbs, we invited participants to introspect about what the destination might believe about the proverbs (e.g., “What do you think that the celebrities would think about the proverbs?”). Group comparisons demonstrated better destination memory after the introspection than when no introspection was implemented in control participants, but there were no significant differences between the two conditions in patients with TBI. However, analyses of individual profiles demonstrated that more than half (*n* = 13) of the patients with TBI demonstrated better destination memory after introspection. While these results demonstrate a beneficial effect of introspection on destination memory for some cases of patients with TBI, more research is needed to reveal how introspection may influence patients’ memory in social interactions.

## 1. Introduction

A key consequence of traumatic brain injury (TBI) is changes in social cognition, resulting in indifference to other people’s feelings, poor social judgment and communication, emotional instability, or impulsivity. These social dysfunctions are common in TBI and can have serious consequences for patients’ daily life activities [1,2]. While survivors of TBI may succeed in maintaining meaningful social relationships and even returning to work, others fail to maintain social relationships [3] even many years after the injury [4]. For relatives of patients with TBI, these social impairments can be a higher burden than the cognitive or even physiological impairments [5]. Impairments in social cognition in TBI have also been reported by research on destination memory. Destination memory refers to the ability to remember to whom a piece of information was previously transmitted [6,7,8,9,10,11,12,13,14,15,16,17,18,19,20,21,22,23,24,25]. Research has demonstrated a decline in destination memory in TBI, which was found to be related to the ability to represent, conceptualize, and reason about others’ mental states [11,18,26]. Building on this research, we investigated whether introspecting about the destination’s mental state would improve destination memory in patients with TBI. We expected positive effects of introspection on destination memory in patients with TBI.

A decline in destination memory has been reported in TBI. This decline was reported by Wilu Wilu, Coello [26], who invited patients with TBI and control participants to tell proverbs to pictures of celebrities. Participants were invited to indicate to which celebrity they had previously told the proverbs. Besides the assessment of destination memory, participants performed a binding control task in which they were invited to associate letters with their corresponding location. Results demonstrated lower destination memory and binding in patients with TBI than in control participants. Furthermore, in both populations, significant correlations were observed between destination memory and binding. According to Wilu Wilu and Coello [26], these results demonstrate difficulties in attributing information to its appropriate destination in patients with TBI, probably due to difficulties in binding separate information together to form an episodic event or even owing to the general decline in episodic memory in TBI [27,28]. A decline in destination memory has also been reported in a study by Wilu Wilu and Allain [18], who also reported a relationship between this decline and the cognitive theory of mind. These results demonstrate a relationship between the decline in destination memory in patients with TBI and difficulties in inferring and predicting the cognitive states of interlocutors. The relationship between destination memory and theory of mind has been reported in TBI as well as in normal aging and Alzheimer’s disease [29,30]. According to this research, when telling information to interlocutors in social interactions, we mainly focus on, observe, and evaluate their feedback. This interlocutor-monitoring process is closely related to the theory of mind since both abilities imply processing the cognitive states of others, which may explain why high values in the theory of mind may result in a better destination memory. Together, research has demonstrated a decline in destination memory in patients with TBI, which is associated with a decline in their ability to monitor and infer the thoughts, cognitive states, and intentions of interlocutors.

If the decline in destination memory is associated with a decline in the ability to monitor and infer cognitive states of interlocutors, then probing processing of these states may improve destination memory. This issue was investigated by El Haj, Allain [24], who evaluated whether a decreased destination memory may be observed when patients with Alzheimer’s disease were asked to process attributes of the destinations. More specifically, the authors conducted tests on two groups: patients with Alzheimer’s disease and healthy older adults, under two different conditions. In the first condition, which is the typical assessment of destination memory, participants were asked to share proverbs with the faces of celebrities. Later, in a recognition test, they had to determine whether they had previously shared a particular proverb with that specific celebrity or not. In the second condition, the same procedures were followed, but with an additional step. After sharing the proverbs, participants were prompted to introspect about what the celebrities might think about those proverbs. For example, they were asked, “What do you think the celebrity would think about that proverb?”. Results demonstrated that introspection had a positive impact on destination memory in healthy older adults. However, this effect was not observed in patients with Alzheimer’s disease. Nevertheless, a closer examination of individual profiles showed that almost half of the patients with Alzheimer’s disease exhibited improved destination memory after introspection. These findings demonstrate the beneficial effect of introspection on destination memory in the context of normal aging and, to some extent, in certain patients with Alzheimer’s disease. Based on these findings, we investigated whether introspection would improve destination memory in patients with TBI.

To summarize, a key consequence of TBI is changes in social cognition, including a decline in the theory of mind [31,32,33,34,35]. Because a decline in social cognition can yield serious consequences for patients’ daily lives, studies in the field of social cognition may yield positive outcomes for the improvement in the quality of life of patients with TBI. Destination memory is, therefore, a prominent area of research in TBI as, beyond its associations with episodic memory [36], this memory system is intimately associated with social cognition [9,10,11]. Previous research has demonstrated a decline in destination memory in TBI [26]. However, this research did not investigate whether introspection would improve destination memory in patients with TBI. We thus investigated whether introspecting about the destination’s mental state (e.g., what the destination might think about the transmitted information?) would improve destination memory in patients with TBI. Because previous research has demonstrated the beneficial effect of introspection on destination memory in normal aging, and at least in some patients with Alzheimer’s disease [24], we expected positive effects of introspection on destination memory in patients with TBI.

## 2. Methods

### 2.1. Participants

The current study included 24 participants with TBI and 25 control participants (demographic characteristics are provided in Table 1). All participants provided written and informed consent, and the study was carried out in accordance with the principles laid down by the Helsinki Declaration. Patients were recruited from neurological centers in the region of Lille, France. Data were collected in one session during the period of 2017–2020, and the sample size was the maximum number of participants who could be enrolled during this period. For all participants, exclusion criteria were a history of neurological (other than TBI) or psychiatric condition and a history of substance abuse/dependence. The detailed characteristics of TBI are provided in Table 2. The patients with TBI were at least six months post-injury and had suffered post-traumatic amnesia as reported in their medical records or obtained using careful clinical questioning of the participant and/or a medical physician. All patients suffered severe TBI, as demonstrated using their scores on the Glasgow Coma Scale (less than eight points), which was rated in the emergency room or at the scene of the accident. The heterogeneity of lesions in our sample, as shown in Table 2, is typically observed in this population [37,38].

### 2.2. Cognitive Function

We evaluated the overall cognitive function of the participants using the Montreal Cognitive Assessment [39]. This widely utilized assessment tool encompasses 30 items designed to screen various cognitive domains, including attention, orientation, language, verbal memory, visuospatial abilities, and, to some extent, executive functions like flexibility and planning. To assess episodic memory, we employed the selective reminding task of Grober and Buschke [40], as this task has been validated in French populations [41]. During this task, participants were tasked with memorizing 16 words, each representing a different item (e.g., guitar) from distinct semantic categories (e.g., musical instruments). The immediate cued recall was followed by a distraction phase, during which participants were required to count backward from 374 within a 20-s timeframe. This distraction phase was succeeded by a two-minute period of free recall, and the score obtained from this phase served as a measure of episodic recall (with a maximum score of 16 points). As indicated in Table 1, patients with traumatic brain injury (TBI) exhibited lower general cognitive function and episodic memory compared to the control participants. Note that we assessed only general cognitive function and episodic memory to avoid fatigability and distractions in the patients.

### 2.3. Procedures

**Figure 1:**
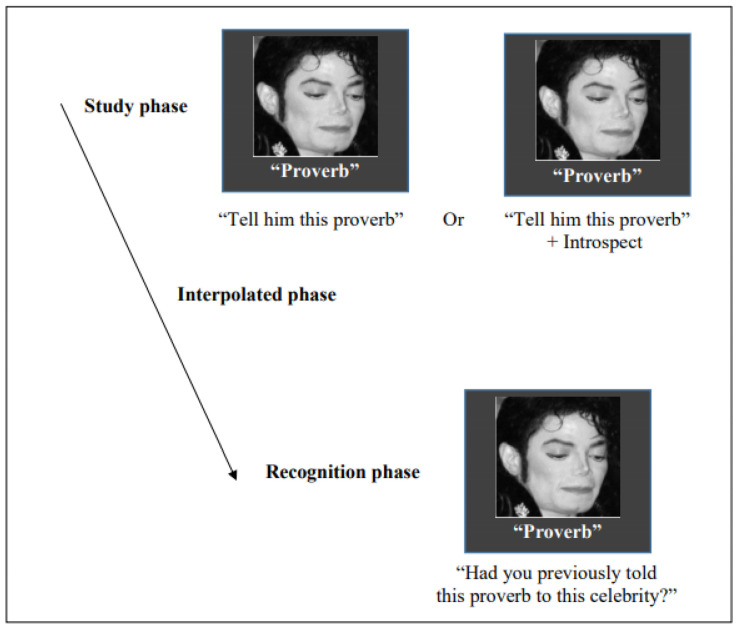
In the destination memory task, participants were tested on two conditions. In the first one, they had to tell proverbs to celebrities’ faces, and after an interpolated phase, they had to decide whether they had previously told that proverb to that celebrity. The same procedures were replicated on the second condition; however, after telling the proverbs, the participants were asked to introspect about what the celebrities might think about the proverbs. *Note: Michael Jackson’s image is covered by Creative Commons copyright*.

Destination memory assessment and response recording were conducted using the software package Psychopy [42] coupled with a laptop computer and a 17-inch LCD display. The procedures, depicted in Figure 1, consisted of a study phase, an interpolated phase (to avoid relying on immediate memory), and a recognition phase. Participants were informed in advance that their memory of the association between proverbs and faces would be subsequently tested. The study phase consisted of 24 trials, each beginning with a one-sec white fixation cross followed by a proverb (e.g., “Fortune favors the bold”) presented in white Times New Roman 40-point font below a (12 × 12 cm) celebrity’s face (e.g., Elvis Presley). During each trial, participants were tasked with sharing a proverb with a celebrity, and there was no imposed time limit for this interaction. It is worth noting that the proverbs used in this study adhered to the formal French language, and those featuring archaic or vernacular language were intentionally excluded. The celebrities chosen for this task encompassed a range of well-known figures, including French and international musicians, artists, politicians, entertainers, athletes, and other prominent individuals who were notable and frequently featured in the news. It is important to mention that in previous research conducted with French populations, careful attention was paid to controlling and standardizing the level of familiarity associated with both the proverbs and the selected celebrities [43].

Following the study phase, there was an interpolated phase in which participants were asked to audibly read strings of three-digit numbers for one minute. This interpolated phase was immediately succeeded by the recognition phase.

In the recognition phase, the 24 proverbs and faces that had been presented during the study phase were paired together and displayed in a random order. This included 12 intact pairs, where the original pairings were maintained, and 12 pairs that were reconfigured into new combinations. Each pair was presented one at a time, with the proverb presented in white Times New Roman 40-point font beneath a celebrity’s face. Participants were tasked with determining, without any time constraints, whether they had previously shared that specific proverb with that particular face. They indicated their response by pressing a green key for “yes” if they had shared the proverb or a red key for “no” if they had not. Following each response, a blank screen was displayed for 250 milliseconds, and then the next test trial commenced.

We replicated the same procedures in the retrospection condition but with a different set of faces and proverbs. Importantly, during the study phase of the introspection condition, immediately after sharing the proverbs, we encouraged participants to introspect about the cognitive states of the individuals receiving these proverbs. To probe this introspection, we posed questions such as “What do you think this person would think about the proverb?” Participants’ responses were then followed by additional questions like “Why do you think that person would have that thought about the proverb?”. In cases where participants were unable to provide answers or responded with “I do not know”, we further inquired, “Do you believe this person would find this proverb interesting?” and subsequently asked, “Why do you think this person would find this proverb interesting?” If participants were still unable to respond to the latter question, no additional prompts were given. Throughout this introspection process, the pictures and proverbs remained visible to the participants.

While the “introspection” and “no introspection” conditions were implemented in the same session, they were randomly counterbalanced across participants and separated by the episodic memory test. Regarding performances, and as recommended for analyzing recognition memory [38], performance refers to the proportion of hits (correct “yes” responses) minus the proportion of false alarms (incorrect “yes” responses).

## 3. Results

**Figure 2:**
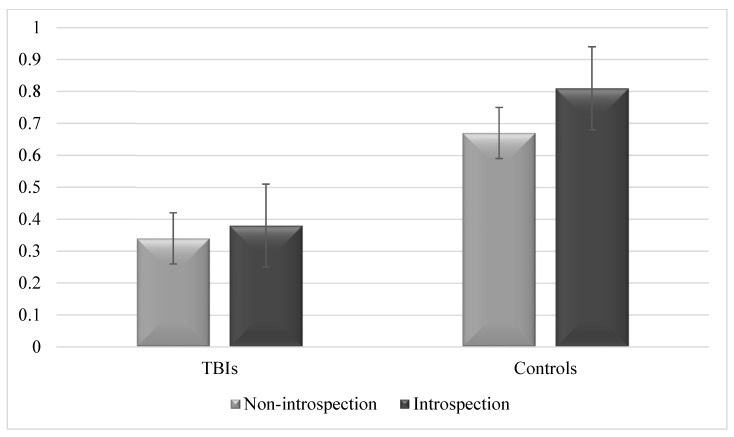
Destination memory performance in patients with TBIs and controls after introspection and when no introspection was implemented at all. *Note: Performance refers to the proportion of hits minus the proportion of false alarms. Error bars are 95% within-subjects confidence intervals*.

We compared scores on destination memory between patients with TBI and controls across the two experimental conditions (non-introspection vs. introspection); performances are depicted in Figure 2. Because of abnormal distributions, demonstrated using the Kolmogorov–Smirnov test, non-parametric tests were used. Results were provided with the observed power as follows: *d* = 0.2 is considered a small effect size, *d* = 0.5 represents a medium effect size, and *d* = 0.8 refers to a large effect size [44]; *d* was calculated for non-parametric tests following recommendations by Rosenthal and DiMatteo [45], and Ellis [46]. For all tests, the level of significance was set as *p* ≤ 0.05. To provide further insight into the introspection effect, group comparisons were followed by an analysis of individual profiles.

### 3.1. Destination Memory Improvement after Introspection in Controls but Not Patients with TBI

While Wilcoxon tests showed no significant differences in destination memory between the “non-introspection” and “introspection” conditions in patients with TBI (*W* = 66.00, *Z* = −1.46, *p* = 0.14, Cohen’s *d =* 0.62), controls showed improved destination memory after introspection (*W* = 37.5, *Z* = −2.52, *p* = 0.012, Cohen’s *d =* 1.17). Mann–Whitney’s U tests demonstrated poor destination memory in patients with TBI than in controls in the “non-introspection” condition (*U* = 87.00, *Z* = −4.29, *p* < 0.001, Cohen’s *d =* 1.55) and “introspection” condition (*U* = 27.00, *Z* = −5.51, *p* < 0.001, Cohen’s *d =* 2.55).

### 3.2. Individual Profiles: More Benefits Than Decline in Destination Memory after Introspection in All Participants

To establish a threshold for destination memory performance in patients with Traumatic Brain Injury (TBI) after the introspection process, we standardized the performances by converting them into Z-scores, using the mean and standard deviations derived from the control group data. Performance was significantly affected if the Z-scores fell below -2 standard deviations from the control group’s mean. Analysis revealed that in the “no introspection” condition, the performance of four TBI patients dipped below this cut-off, whereas in the “introspection” condition, only one patient’s performance fell below this threshold. To gain further insight into individual profiles, we counted, compared with the “no introspection” condition, the number of participants who showed (1) increased destination memory after introspection, (2) decreased destination memory after introspection, and (3) similar performance across the two conditions. In patients with TBI, introspection resulted in more improvement (*n* = 13) than decline (*n* = 9) or similar performance (*n* = 2) [χ^2^ (2, *N* = 24) = 7.75, *p* = 0.02, Cohen’s *d =* 1.38]. In controls, introspection also resulted in more improvement (*n* = 16) than decline (*n* = 4) or similar performance (*n* = 5) [χ^2^ (2, *N* = 25) = 10.64, *p* = 0.005, Cohen’s *d =* 1.72]. Importantly, the patients who demonstrated improvement in destination memory after introspection were P2, P4, P5, P6, P7, P8, P12, P13, P18, P20, P21, P22, P24, those who demonstrated decline in destination memory after introspection were P1, P9, P10, P14, P15, P16, P17, P19 and P23, and those who demonstrated similar performance across the two conditions were P3 and P11.

## 4. Conclusions

Taken together and contrary to our hypothesis, group comparisons demonstrated beneficial effects of introspection only in the control group. However, analysis of individual profiles showed that these benefits were observed in more than half of the patients.

### 4.1. Discussion

We investigated whether introspection would improve destination memory in patients with TBI. Groups-comparisons showed higher destination memory after introspection than when no introspection was implemented in the control participants, but no significant differences for patients with TBI. However, individual profiles showed that more than half of the patients demonstrated better destination memory after introspection.

Destination memory is intimately intertwined with social cognition as remembering to whom information was previously told requires processing attributes of interlocutors. When relaying information to interlocutors, we mainly focus on observing and evaluating their feedback. This monitoring activity allows for inferring intreloculotrs’ mental states and, consequently, in-depth encoding of the destination and improvement in destination memory. This assumption can be supported by research demonstrating a relationship between destination memory and the ability to introspect about others’ mental states (i.e., theory of mind) in normal aging [30], Alzheimer’s Disease [29], and in patients with TBI [18]. This assumption can be further supported by research demonstrating better destinations after introspection in the general population [24], which mirrors the performances of our control participants. However, although group comparisons demonstrated no significant differences between the “introspection” and “no-introspection” conditions in patients with TBI, individual profiles showed that more than half of patients demonstrated better destination memory after introspection. Interestingly, when looking at the individual characteristics of these patients (see Table 2), we can remark on a heterogeneity of the etiology and pathophysiology of their traumas. The heterogeneity can be considered as the hallmark of TBI [47]. Thus, the improvement in destination memory after introspection in the 13 patients with TBI seems not to be related to the etiology and pathophysiology of traumas. The same thing can be said for patients who demonstrated a decline in destination memory after introspection and those who demonstrated similar performance across the “introspection” and “no-introspection” conditions. While our findings mirror the general neurological and cognitive heterogeneity of TBI [37,38], they demonstrate that some patients with TBI can benefit from introspection to improve their memory performances. TBI are heterogeneous in nature, and patients with TBI have significant variations in the location and severity of injury, resulting in a significant variation in cognitive performances and insight [33,48,49,50]. This cognitive variation may explain why some patients with TBI may benefit from introspection to improve destination memory while others fail to do so.

By demonstrating some benefits of introspection on memory (at least for some patients), our study paves the way for research on the relationship between memory and social cognition in TBI. Previous research has extensively evaluated memory in TBI, demonstrating a decline in episodic memory [51,52,53,54] and working memory [55,56,57]. Further, research has extensively evaluated social cognition in TBI. Research in this area has demonstrated difficulties for patients with TBI to recognize emotions as expressed by faces, voices, or body postures [58,59,60]. Research has also demonstrated a significant decline in theory of mind in patients with TBI, as observed with classic stories based on false beliefs or understanding a faux pas [31,33,61,62,63]. In a similar vein, patients with TBI tend to demonstrate difficulties in predicting the intentions of characters in cartoon sequences [64] as well as a person’s mental states based on the eye region of the face [64,65,66]. Besides impairment of emotion processing and theory of mind, patients with TBI demonstrate linguistic and communication difficulties that hinder social interactions [67]. The wealth of research on social cognition in TBI can be attributed to the fact that difficulties in social functioning are common following TBI and have negative consequences on daily life and reintegration into society [68,69,70]. Furthermore, while physical and motor symptoms may stabilize over time following injury, social disturbances cause the greatest long-term distress [71,72]. Together, there is a wealth of research on social cognition and memory functioning in TBI; however, little attempt has been made to investigate the relationship between social cognition and memory in TBI. The present paper addresses this challenge by examining how introspecting interlocutors’ mental states may influence memory in TBI.

### 4.2. Destination Memory and Social Cognition in TBI

Compared with other memory systems (e.g., episodic memory, working memory), destination memory is essentially oriented toward social interactions [9,10,11,13,14,17,19,23]. Supporting this assumption, research has demonstrated how destination memory can vary following the familiarity of interlocutors [43] or their age [73]. Interestingly, research has demonstrated how destination memory can vary following emotion as expressed by interlocutors [74,75]. It would be of interest to evaluate whether patients with TBI can successfully introspect the emotional characteristics of their destination and, consequently, whether such introspection may improve their destination memory. Further, research has assessed the effects of social stereotypes on destination memory [76]. This research has demonstrated increased destination memory for consistent facts (e.g., facts concerning medicine that were previously told to a physician) than for inconsistent facts (e.g., facts concerning medicine that were previously told to a mechanic). It would, therefore, be of interest to investigate whether TBI can build on these (in)consistencies to improve their destination memory. This research may further demonstrate how social cognition may influence memory in patients with TBI.

### 4.3. Limitations and Perspectives

One limitation of this paper may be the relatively small sample size, as our study did not have sufficient statistical power to detect significant differences between patients with TBI and controls on the “introspection” condition. While recruiting patients with TBI is challenging, future research may replicate our experimental design in a larger sample. Regardless of its potential limitations, our study paves the way for a novel line of research on the effects of processing interlocutors’ mental states on the general ability of patients with TBI to remember interlocutors. Building on this research, clinical rehabilitation programs may be able to target the relationship between social cognition and memory in patients with TBI, for instance, how the rehabilitation of the ability to ascribe cognitive and affective states to others and infer their intentions from feedback (i.e., rehabilitation theory of mind) may improve the patient’s memory in social interactions.

### 4.4. Conclusions

This study investigates the effects of introspection on destination memory in both control participants and patients with TBI. While introspection appeared to improve destination memory in some patients with TBI, this effect was not observed in all the patients, with some even demonstrating lower destination memory after introspection. Further research is required to fully understand the mechanisms and broader implications of introspection on memory, especially in social contexts. Critically, and by considering the heterogeneity of TBI, this research should implement individual profile analysis to tailor interventions and support to the specific needs and circumstances of each patient.

## Figures and Tables

**Table 1:** Demographic and cognitive characteristics for patients with Traumatic Brain Injury (TBI) and control participants.

	TBI *n* = 24	Controls *n* = 25	
Gender (F/M)	15/9	15/10	χ^2^(1, N = 49) = 0.03, *p* = 0.85
Age in years	34.40 (9.28)	32.12 (8.03)	*t*(47) = 0.15, *p* = 0.88
Years of formal education	12.60 (2.86)	13.16 (2.73)	*t*(47) = 0.03, *p* = 0.98
General cognitive function	21.02 (6.32)	28.25 (0.96)	*t*(47) = 5.65, *p* < 0.001
Episodic memory	7.66 (4.64)	12.01 (2.24)	*t*(47) = 4.21, *p* < 0.001

Note: Standard deviations are provided in parenthesis; general cognitive function was assessed with the Montreal Cognitive Assessment, and the maximum score was 30 points; episodic memory was assessed with the selective reminding task, and the maximum score was 16 points.

**Table 2:** Individual characteristics of Traumatic Brain Injury.

Patient	Etiology	Glasgow Coma Scale	Post Traumatic Amnesia	Acute CT-Scan Findings
1	MVA	6	One day	Right frontal epidural hemorrhage, subarachnoid hemorrhage, multiple fractures
2	Fall	4	A few minutes	Left subarachnoid hemorrhage
3	Assault	5	Four-five hours	Occipital skull fracture
4	NVA	4	Two days	Hemorrhagic contusions
5	Fall	7	Three-five min	Hemorrhagic contusions
6	Fall	7	One day	Subarachnoid hemorrhage
7	Assault	7	A few minutes	Frontal skull fracture
8	Fall	5	One day	Basilar skull fracture
9	MVA	2	Duration unclear	Temporal and parietal fractures and contusions, right subdural hemorrhage
10	Assault	4	Two days	Occipital skull fracture
11	Fall	3	A few minutes	Bifrontal contusions (required craniotomy)
12	Fall	7	Duration unclear	Left temporal bone fracture (required craniotomy)
13	NVA	5	Eight days	Subarachnoid hemorrhage
14	Fall	6	Three-four min	Subdural hemorrhage
15	Fall	7	Two days	Intracranial hemorrhage (requires craniotomy)
16	Fall	4	Several hours	Subarachnoid hemorrhage
17	MVA	3	Two days	Epidural hemorrhage, right temporal bone fracture (required craniotomy)
18	NVA	7	Duration unclear	Temporal and parietal fractures and contusions
19	Fall	5	Ten days	Intracranial hemorrhage (requires craniotomy)
20	Fall	2	One-two hours	Subarachnoid hemorrhage, occipital skull fracture
21	Fall	7	Two days	Subarachnoid hemorrhage
22	Assault	6	A few minutes	Right temporal bone fracture (required craniotomy)
23	Fall	5	One day	Subdural hemorrhage
24	Assault	7	A few minutes	Subarachnoid hemorrhage

Note: MVA = motored vehicle accident; NVA = non-motored vehicle accident.

## Data Availability

Raw data are available upon request to the corresponding author.
